# Antimicrobial resistance in intensive care patients hospitalized with SEPSIS: a comparison between the COVID-19 pandemic and pre-pandemic era

**DOI:** 10.3389/fmed.2024.1355144

**Published:** 2024-05-15

**Authors:** Katia Falasca, Luigi Vetrugno, Paola Borrelli, Marta Di Nicola, Claudio Ucciferri, Alessandra Gambi, Magdalena Bazydlo, Giorgia Taraschi, Jacopo Vecchiet, Salvatore Maurizio Maggiore

**Affiliations:** ^1^Clinic of Infectious Diseases—Department of Medicine and Science of Aging, “G. d’Annunzio” University Chieti-Pescara, Chieti, Italy; ^2^Department of Anesthesiology, Critical Care Medicine and Emergency—Department of Medical, Oral and Biotechnological Sciences, “G. d’Annunzio” University Chieti-Pescara, Chieti, Italy; ^3^Laboratory of Biostatistics, Department of Medical, Oral and Biotechnological Sciences, “G. d’Annunzio” University Chieti-Pescara, Chieti, Italy; ^4^Laboratory of Clinical Pathology, SS Annunziata Hospital, Chieti, Italy; ^5^Department of Anesthesiology, Critical Care Medicine and Emergency, Department of Innovative Technologies in Medicine and Dentistry, “G. d’Annunzio” University Chieti-Pescara, Chieti, Italy

**Keywords:** COVID-19, sepsis, ICU, mortality, antimicrobial therapy

## Abstract

**Introduction:**

Coronavirus disease 2019 (COVID-19) is a highly contagious viral illness caused by severe acute respiratory syndrome coronavirus 2 (SARS-CoV-2). It has had a dramatic effect on the world, resulting in millions of deaths worldwide and causing drastic changes in daily life. A study reported that septic complications were associated with high mortality in COVID-19 patients. This study aimed to evaluate how the COVID-19 pandemic changed the pre-pandemic and post-pandemic prevalence of sepsis in ICUs and to evaluate the different risk factors associated with mortality and the different diffusion of microorganisms and their resistance.

**Materials and methods:**

We conducted a single-center retrospective observational clinical study, observing all patients in the ICU of the SS Annunziata Hospital in Chieti (Italy) who were diagnosed with sepsis and had a bacterial isolate from their blood culture. Sepsis was diagnosed by SEPSIIS III criteria. We enrolled all in-patients in the ICU from January 2018 to December 2021. We divided the patients into three groups: (1) non-pandemic period (Np) hospitalized in 2018–2019, (2) pandemic period (Pp)-COVID hospitalized in 2020–2021 with a diagnosis of COVID-19, and (3) Pp-non-COVID patients hospitalized in 2020–2021 without a diagnosis of COVID-19.

**Results:**

From January 2018 to December 2021, 1,559 patients were admitted to the ICU, of which 211 patients [36 (17.1%) in 2018, 52 (24.6%) in 2019, 73 (34.6%) in 2020, and 50 (23.7%) in 2021, respectively] met the selection criteria: 88 patients in period Np, 67 patients in Pp without COVID-19, and 56 patients Pp with COVID-19. The overall mortality of these patients was high (65.9% at 30 days in Np), but decreased during the Pp (60.9%): Pp-non-COVID was 56.7% vs. Pp-COVID 66.1%, with a statistically significant association with APACHE III score (OR 1.08, 95%CI 1.04–1.12, *p* < 0.001), SOFA score (OR 1.12, 95%CI 1.03–1.22, *p* = 0.004), and age (OR 1.04, 95%CI 1.02–1.07, *p* < 0.0001). Between the Np vs. Pp periods, we observed an increase in a few Gram-positive bacteria such as *S. capitis* (1 pt. −0.9% vs. 14 pt. −7.65%- *p* = 0.008), *S. epidermidis*, *Streptococcus* spp., and *E. faecalis*, as well as a decrease in a case of blood culture positive for *S. aureus*, *S. hominis*, *and E. faecium.* In Gram-negative bacteria, we observed an increase in cases of *Acinetobacter* spp. (Np 6 pt. −5.1%- vs. Pp 20 pt. −10.9%, *p* = 0.082), and *Serratia* spp., while cases of sepsis decreased from *E. faecium* (Np 11 pt. −9.4%- vs. Pp 7 pt. −3.8%, *p* = 0.047), and *Enterobacter* spp.*, S. haemolyticus*, *S. maltophilia*, *Proteus* spp., and *P. aeruginosa* have not changed. Finally, we found that resistance to OXA-48 (*p* = 0.040), ESBL (*p* = 0.002), carbapenems (*p* = 0.050), and colistin (*p* = 0.003) decreased with time from Np to Pp, particularly in Pp-COVID.

**Conclusion:**

This study demonstrated how the COVID-19 pandemic changed the prevalence of sepsis in the ICU. It emerged that the risk factors associated with mortality were APACHE and SOFA scores, age, and, above all, the presence of ESBL-producing bacteria. Despite this, during the pandemic phase, we have observed a significant reduction in the emergence of resistant germs compared to the pre-pandemic phase.

## Introduction

Coronavirus disease 2019 (COVID-19) is a contagious viral disease caused by severe acute respiratory syndrome coronavirus 2 (SARS-CoV-2). It has had a dramatic effect on the world, causing almost 7 million deaths worldwide and changing daily life. COVID-19 has many reports concerning different clinical manifestations and different risk factors and biomarkers associated with the worsening (1–3). According to a report from the end of May 2020, 1.3 million cases were reported to the United States Centers for Disease Control and Prevention (CDC), with 14% requiring hospitalization, 2% admitted to the intensive care unit (ICU), and 5% dying. The individual risk of severe illness varies by age, underlying comorbidities, and vaccination status. Sepsis is one of the leading causes of death associated with SARS-CoV-2 infection, accounting for 65% (4).

A 2016 SCCM/ESICM task force has defined sepsis as life-threatening organ dysfunction caused by a dysregulated host response to infection (Sepsis-3) as evidenced by organ dysfunction and infection (5).

The Global Burden of Disease Study in 2017 reported an estimated 48.9 million incident cases of sepsis (6). Approximately 11 million deaths were reported, representing 19.7% of all global deaths. Overall mortality decreased by almost 53% between 1990 and 2017.

The importance of identifying risk factors for sepsis was highlighted in one epidemiologic study, which found that septic shock was the fifth leading cause of years of lost productive life due to premature mortality (6). Sepsis risk factors include ICU admission (approximately 50% of ICU patients have a nosocomial infection), advanced age (≥65 years), bacteremia, immunosuppression, diabetes and obesity, cancer, previous hospitalization, genetic factors, community-acquired pneumonia, and severe acute respiratory illness from SARS-CoV-2. COVID-19 can predispose people to sepsis from secondary infections (7–10).

A study reported that sepsis, occurring as a complication of COVID-19, was associated with high mortality in COVID-19 patients (11).

Ventilated COVID-19 patients often receive multiple antibiotic courses. At the height of the pandemic, antibiotic stewardship policies were overridden (12), and ICU capacity was increased. A Spanish hospital reported increased antibiotic use (13). Such data raise concerns that resistance in hospitals may increase as a result of COVID-19 pressures, notwithstanding a lack of evidence that this has occurred.

This study aimed to evaluate how the COVID-19 pandemic changed the pre-pandemic and post-pandemic prevalence of sepsis in ICUs and to evaluate the different risk factors associated with mortality and different diffusions of microorganisms and their resistance.

## Methods

### Study design, setting, and population

We carried out a single-center retrospective observational clinical study that observed all the patients in the ICU of the SS Annunziata Hospital in Chieti (Italy) who were diagnosed with sepsis and who presented a bacterial isolate from blood culture.

We enrolled all in-patients in the ICU from January 2018 to December 2021. We divided the patients into three groups: (1) non-pandemic period (Np) hospitalized in 2018–2019, (2) pandemic period (Pp)-COVID hospitalized in 2020–2021 with a diagnosis of COVID-19, and (3) Pp-non-COVID patients hospitalized in 2020–2021 without a diagnosis of COVID-19.

Inclusion criteria:

1. all patients admitted to intensive care for more than 48 h;
2. age over 18 years old; and
3. presence of two or more positive blood cultures in patients with clinical signs of active infection and sepsis.

Exclusion criteria:

1. admission to the ICU for ongoing sepsis;
2. the presence of only one positive blood culture kit; and
3. pregnant women.

The study protocol was approved by the Ethics Internal Committee at the University “G. d’Annunzio” Chieti-Pescara (Ethics Committee Project No. 02 02/02/2022) and was performed according to the ethical standards established in the 1964 Declaration of Helsinki.

### Variables, data sources, and measurement

Demographic data such as age and gender, as well as the presence of comorbidities such as diabetes, active malignancies, chronic kidney disease (CKD), drug addiction, and immunodeficiency were analyzed. Acute Physiologic Assessment and Chronic Health Evaluation (APACHE) score and Sequential Organ Failure Assessment (SOFA) score were calculated for all patients (14).

The days of hospitalization and the presence and type of the isolated germ were evaluated, with the characteristics of resistance and antibiotic therapy carried out empirically and after susceptibility testing.

Sepsis was diagnosed by SEPSIIS III criteria (15) and EUCAST guidelines.

### Antimicrobial susceptibility testing

Every blood culture was placed in a BacT/ALERT® BPA (bioMérieux), which provided both a microbial detection system and a culture media with suitable nutritional and environmental conditions for organisms that might be present in the test sample. Inoculated bottles were incubated in the instrument and continuously monitored for the presence of microorganisms that would grow in the BacT/ALERT BPA bottles. The antimicrobial agents tested included ampicillin, amoxicillin/clavulanic acid, piperacillin/tazobactam, cefuroxime, cefotaxime, ceftriaxone, ceftazidime, cefepime, imipenem, ertapenem, gentamicin, amikacin, ciprofloxacin, levofloxacin, and trimethoprim/sulfamethoxazole. The results were interpreted by the EUCAST guidelines. Extended-spectrum beta-lactamase (ESBL) production was confirmed phenotypically using a combination disk test according to the EUCAST guidelines. Phenotypic screening for carbapenemase production in Enterobacteriaceae was performed using the Carba NP test. An antimicrobial sensitivity test was performed by Vitek 2 (bioMérieux).

### Statistical analysis

Descriptive analysis was carried out using mean and standard deviation (±SD) or median and interquartile range (IQR) for the quantitative variables and percentage values for the qualitative ones. Normality distribution for quantitative variables was assessed by the Shapiro–Wilk test. The association between groups and explicative variables was investigated by Pearson χ2 test and Analysis of Variance (ANOVA) or analog test non-parametric Kruskal Wallis’s test followed by the appropriate post-hoc test if significant. Bonferroni’s correction for multiple comparison tests was applied. The prevalence of patients admitted for infection per year with 95% confidence intervals (CIs) was calculated. In addition, the occurrence of mortality per year was also estimated. Crude odds ratios (ORs) and corresponding 95% CI were calculated to quantify the risk associated with mortality as an explicative variable using the Wald test. Statistical significance was set at a level of ≤0.05, unless adjustments for multiple comparisons were required. All analyses were performed using Stata software v17.1 (StataCorp, College Station, Texas, United States).

## Results

**Figure 1:**
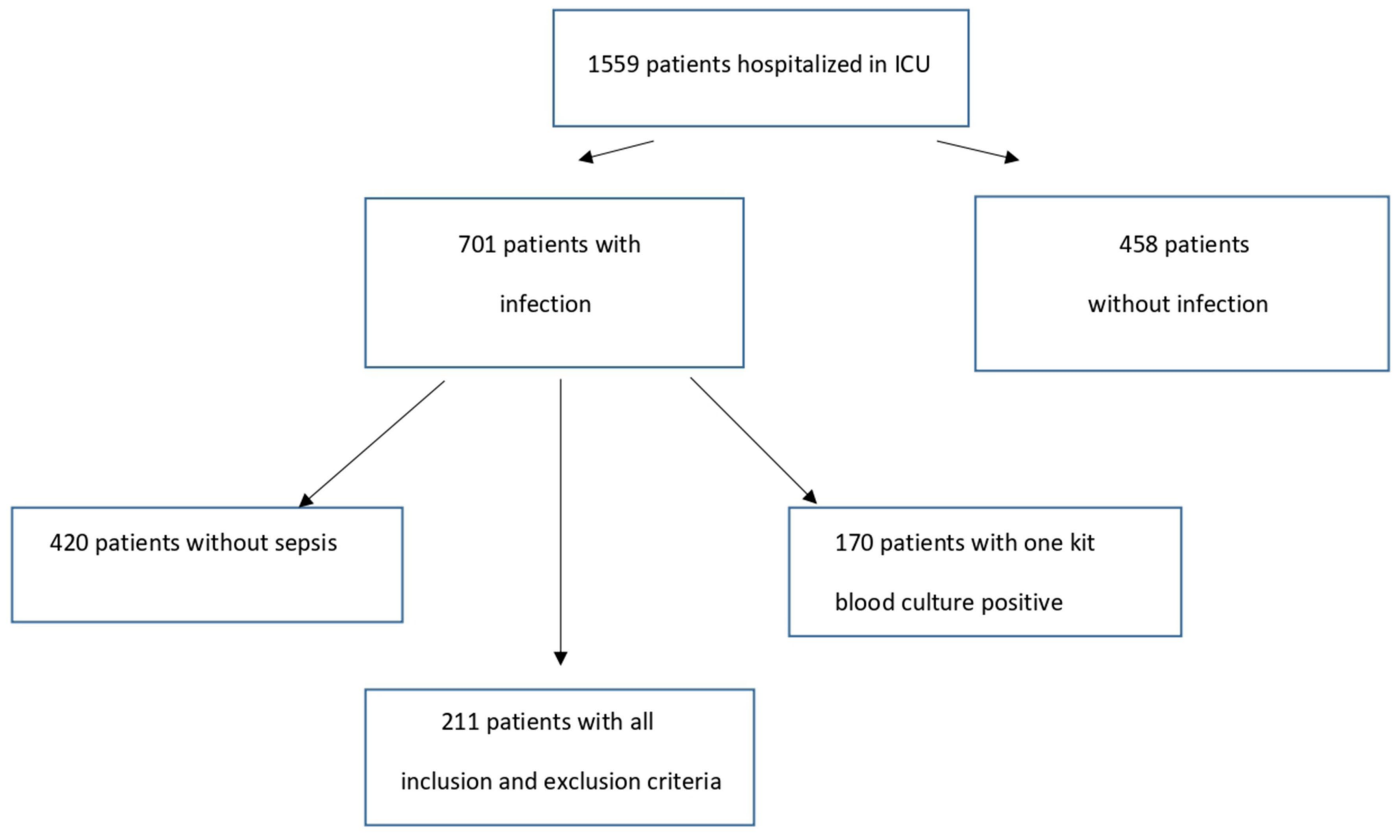
Study flow chart.

From January 2018 to December 2021, 1,559 patients were admitted to the ICU, and 211 patients satisfied the selection criteria [36 (17.1%) in 2018, 52 (24.6%) in 2019, 73 (34.6%) in 2020, and 50 (23.7%) in 2021, respectively]: 88 patients in period Np, 67 patients in Pp without COVID-19, and 56 patients in Pp with COVID-19 (Figure 1). The demographic characteristics of the overall study population and their comorbidities are reported in Table 1. Briefly, the patient’s median age was 70 (IQR 62–78) years, of which 65.6% were male. We found significant differences in the mean score of the SOFA score between the three groups (*p* = 0.028). Specifically, the SOFA score was higher in Pp-COVID 12.2 (±3.46) vs. Np 10.3 (±3.8) with *p* = 0.008.

**Table 1:** Characteristics of sample.

	Total	Np	Pp-non-COVID	Pp-COVID	
	*N* = 211	*N* = 88	*N* = 67	*N* = 56	*p*-value
**Sex, *n*(%)**					
*Female*	72 (34.1%)	32 (36.4%)	23 (34.3%)	17 (30.4%)	0.759
*Male*	139 (65.9%)	56 (63.6%)	44 (65.7%)	39 (69.6%)	
Age, years	70 (62–78)	71 (60.5–80.5)	70 (62–77)	69 (64–75)	0.845
**Comorbidity, *n*(%)**					
*No*	15 (7.1%)	3 (3.4%)	4 (6.0%)	8 (14.3%)	0.056
*Yes*	196 (92.9%)	85 (96.6%)	63 (94.0%)	48 (85.7%)	
**Diabetes mellitus, *n*(%)**					
*No*	163 (77.3%)	64 (72.7%)	53 (79.1%)	46 (82.1%)	0.563
*Yes*	48 (22.7%)	24 (26.3%)	14 (20.9%)	10 (17.9%)	
**Cancer**					
*No*	183 (86.7%)	75 (85.2%)	56 (83.6%)	52 (92.9%)	0.276
*Yes*	28 (13.3%)	13 (14.8%)	11 (16.4%)	4 (7.1%)	
**Acute renal failure, *n*(%)**					
*No*	172 (81.5%)	69 (78.4%)	54 (80.6%)	49 (87.5%)	0.381
*Yes*	39 (18.5%)	19 (21.6%)	13 (19.4%)	7 (12.5%)	
**Drug addiction, *n*(%)**					
*No*	203 (96.2%)	82 (93.2%)	65 (97.0%)	56 (100.0%)	0.103
*Yes*	8 (3.8%)	6 (6.8%)	2 (3.0%)	0 (0.0%)	
**HIV, *n*(%)**					
*No*	202 (95.7%)	83 (94.3%)	63 (94.0%)	56 (100.0%)	0.184
*Yes*	9 (4.3%)	5 (5.7%)	4 (6.0%)	0 (0.0%)	
SOFA Score	11.2 (3.7)	10.6 (3.8)^*^	11.2 (3.6)	12.2 (3.4)^*^	0.028
APACHE Score	25.9 (8.2)	27.3 (8.9)	25.1 (7.5)	24.9 (7.6)	0.122
ICU, days	18 (7–35)	14 (3–37)	19 (9–38)	18 (10–31)	0.293

*N* (%) mean and (sd) or median and interquartile range (IQR) are shown when appropriate. ^*^p-value < α/3 for Bonferroni multiple testing correction vs. Np.

The APACHE score remained unchanged significantly throughout the observation period. In the Pp group, patients remained longer in the ICU for 19 (9–38) days compared to 14 (3–37) days in the Np group (*p* = 0. 293).

**Figure 2:**
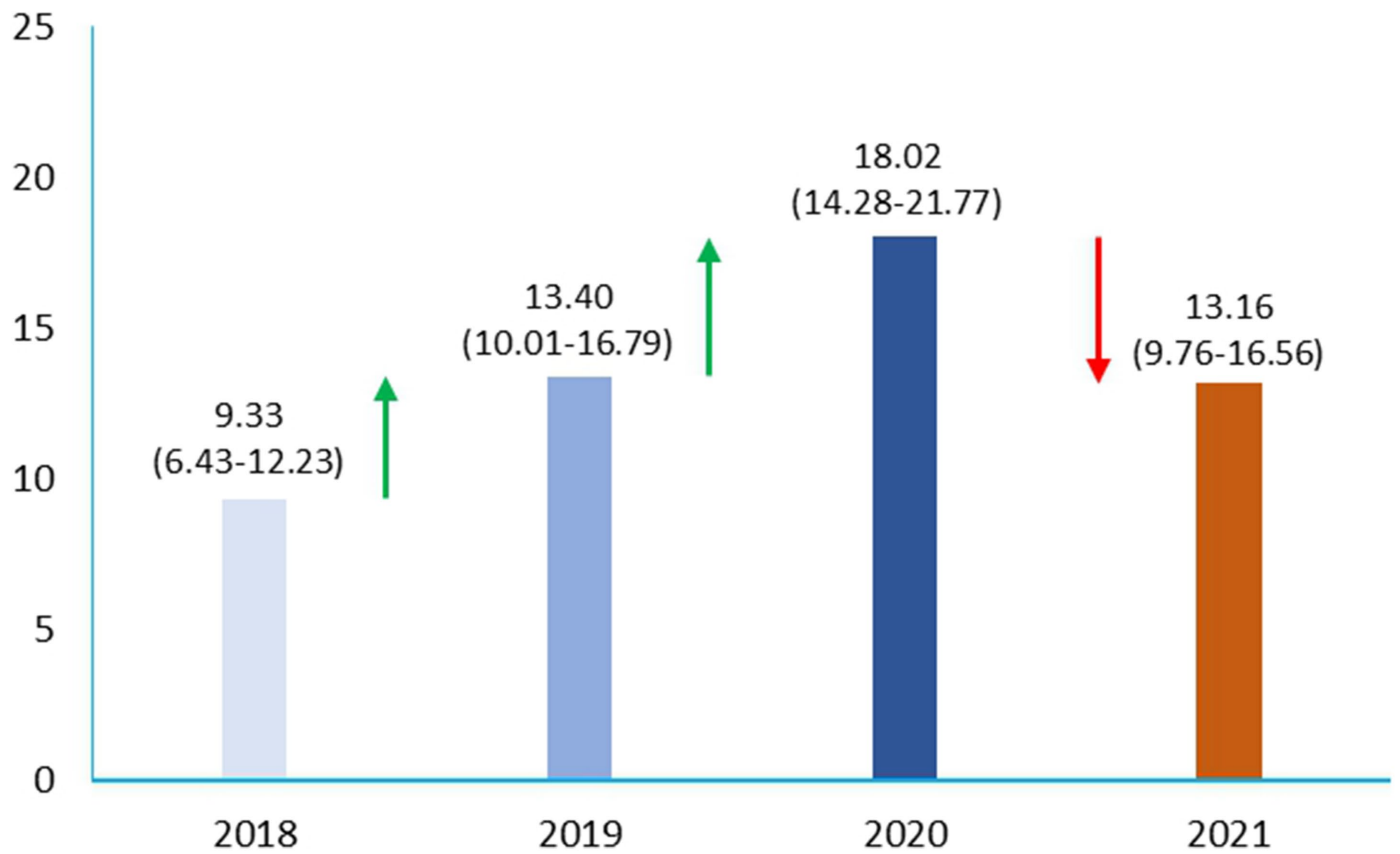
Prevalence of sepsis shock in pre-pandemic and pandemic era in ICU.

In the two study periods, with our strict criteria, Np and Pp sepsis showed an overall prevalence of 13.53%, with data of 9.33% in 2018, 13.40% in 2019, 18.02% in 2020, and 13.16% in 2021, respectively (Figure 2).

We found that among patients enrolled in the study, 135 (64%) had sepsis of medical origin and 76 (36%) had sepsis of surgical origin. However, the overall mortality rate for these patients was high, 65.9% in 30 days, but mortality decreased during the Pp to 60.9%: Pp-non-COVID was 56.7%, compared to Pp-COVID of 66.1%. Furthermore, in the overall population, ESBL microorganisms were associated with increased mortality (68% vs. 55%, with p = 0.028).

Crude OR indicates that the occurrence of mortality increased with the SOFA score (OR 1.12, 95%CI 1.03–1.22, *p* = 0.004), APACHE score (OR 1.08, 95%CI 1.04–1.12, *p* < 0.0001), and age (OR 0.98, 95%CI 0.97–0.99, *p* = 0.023; Table 2).

**Table 2:** Crude OR and 95% CI for identifying risk associated with mortality.

	^*^ORc (95% CI)	*P-*value
**Sex**		
*Female*	1	
*Male*	0.86 (0.47–1.57)	0.631
**Groups**		
*Np*	1	
*Pp-non-COVID*	0.49 (0.25–0.96)	0.039
*Pp-COVID*	0.73 (0.35–1.50)	0.396
Age, years	1.04 (1.02–1.07)	<0.0001
**Comorbidity**		
*No*	1	
*Yes*	1.31 (0.44–3.84)	0.619
**Diabetes mellitus**		
*No*	1	
*Yes*	0.89 (0.45–1.77)	0.757
**Cancer**		
*No*	1	
*Yes*	0.65 (0.28–1.46)	0.298
**Acute renal failure**		
*No*	1	
*Yes*	1.92 (0.85–4.31)	0.111
**Drug addiction**		
*No*	1	
*Yes*	0.50 (0.12–2.07)	0.343
**HIV**		
*No*	1	
*Yes*	1.03 (0.25–4.27)	0.959
SOFA Score	1.12 (1.03–1.22)	0.004
APACHE Score	1.08 (1.04–1.12)	<0.0001
ICU, days	0.98 (0.97–0.99)	0.023

^*^ORc, crude odds ratio; 95% CI, 95% confidence interval.

### Characteristics of the overall germs in the ICU

The blood cultures from CVC were positive on 23.1% in Np and an increase in the Pp-noCOVID compared with Pp-COVID 67.3% vs. 72.4% (*p* < 0.0001). In addition, we found that resistance to OXA-48 (*p* = 0.040), ESBL (*p* = 0.002), carbapenems (*p* = 0.050), and colistin (*p* = 0.003) decreased with time from Np to Pp, particularly in Pp-COVID (Table 3).

**Table 3:** Characteristics of the overall bacteria in ICU.

	Total	Np	Pp-non-COVID	Pp-COVID	
	*N* = 300	*N* = 117	*N* = 107	*N* = 76	*P-*value
***S. aureus***					
*No*	274 (91.3%)	105 (89.7%)	95 (88.8%)	74 (97.4%)	0.093
*Yes*	26 (8.7%)	12 (10.3%)	12 (11.2%)	2 (2.6%)	
***S. hominis***					
*No*	290 (96.7%)	112 (95.7%)	104 (97.2%)	74 (97.4%)	0.848
*Yes*	10 (3.3%)	5 (4.3%)	3 (2.8%)	2 (2.6%)	
***S. capitis***					
*No*	285 (95.0%)	116 (99.1%)	102 (95.3%)	67 (88.2%)	0.003
*Yes*	15 (5.0%)	1 (0.9%)^*^	5 (4.7%)	9 (11.8%)	
***S. epidermidis***					
*No*	217 (72.3%)	87 (74.4%)	76 (71.0%)	54 (71.1%)	0.821
*Yes*	83 (27.7%)	30 (25.6%)	31 (29.0%)	22 (28.9%)	
**Streptococcus**					
*No*	294 (98.0%)	117 (100.0%)	104 (97.2%)	73 (96.1%)	0.075
*Yes*	6 (2.0%)	0 (0.0%)	3 (2.8%)	3 (3.9%)	
***E. faecalis***					
*No*	286 (95.3%)	113 (96.6%)	103 (96.3%)	70 (92.1%)	0.325
*Yes*	14 (4.7%)	4 (3.4%)	4 (3.7%)	6 (7.9%)	
***E. faecium***					
*No*	282 (94.0%)	106 (90.6%)	101 (94.4%)	75 (98.7%)	0.064
*Yes*	18 (6.0%)	11 (9.4%)	6 (5.6%)	1 (1.3%)	
***E. coli***					
*No*	274 (91.3%)	102 (87.2%)	101 (94.4%)	71 (93.4%)	0.130
*Yes*	26 (8.7%)	15 (12.8%)	6 (5.6%)	5 (6.6%)	
***K. pneumoniae***					
*No*	269 (89.7%)	105 (89.7%)	96 (89.7%)	68 (89.5%)	0.998
*Yes*	31 (10.3%)	12 (10.3%)	11 (10.3%)	8 (10.5%)	
**Proteus**					
*No*	298 (99.3%)	116 (99.1%)	106 (99.1%)	76 (100.0%)	0.709
*Yes*	2 (0.7%)	1 (0.9%)	1 (0.9%)	0 (0.0%)	
**Acinetobacter**					
*No*	274 (91.3%)	111 (94.9%)	97 (90.7%)	66 (86.8%)	0.146
*Yes*	26 (8.7%)	6 (5.1%)	10 (9.3%)	10 (13.2%)	
***S. maltophilia***					
*No*	296 (98.7%)	115 (98.3%)	106 (99.1%)	75 (98.7%)	0.880
*Yes*	4 (1.3%)	2 (1.7%)	1 (0.9%)	1 (1.3%)	
***P. aeruginosa***					
*No*	284 (94.7%)	111 (94.9%)	98 (91.6%)	75 (98.7%)	0.108
*Yes*	16 (5.3%)	6 (5.1%)	9 (8.4%)	1 (1.3%)	
**Enterobacter**					
*No*	290 (96.7%)	112 (95.7%)	105 (98.1%)	73 (96.1%)	0.656
*Yes*	10 (3.3%)	5 (4.3%)	2 (1.9%)	3 (3.9%)	
***S. haemolyticus***					
*No*	293 (97.7%)	112 (95.7%)	107 (100.0%)	74 (97.4%)	0.080
*Yes*	7 (2.3%)	5 (4.3%)	0 (0.0%)	2 (2.6%)	
**Serratia**					
*No*	294 (98.0%)	115 (98.3%)	104 (97.2%)	75 (98.7%)	0.770
*Yes*	6 (2.0%)	2 (1.7%)	3 (2.8%)	1 (1.3%)	
**Peripheral blood cultures**					
*No*	41 (13.7%)	6 (5.1%)	17 (15.9%)	18 (23.7%)	0.001
*Yes*	259 (86.3%)	111 (94.9%)	90 (84.1%)^*^	58 (76.3%)^*^	
**CVC blood cultures**					
*No*	146 (48.7%)	90 (76.9%)	35 (32.7%)	21 (27.6%)	<0.0001
*Yes*	154 (51.3%)	27 (23.1%)	72 (67.3%)^*^	55 (72.4%)^*^	
**Oxa R**					
*No*	82 (27.3%)	23 (19.7%)	32 (29.9%)	27 (35.5%)	0.041
*Yes*	218 (72.7%)	94 (80.3%)	75 (70.1%)^*^	49 (64.5%)^*^	
**ESBL**					
*No*	111 (37.0%)	30 (25.6%)	43 (40.2%)	38 (50.0%)	0.002
*Yes*	189 (63.0%)	87 (74.4%)	64 (59.8%)	38 (50.0%)^*^	
***R Carbapenemi***					
*No*	133 (44.3%)	42 (35.9%)	51 (47.7%)	40 (52.6%)	0.050
*Yes*	167 (55.7%)	75 (64.1%)	56 (52.3%)	36 (47.4%)^*^	
**R Aminoglicosidi**					
*No*	162 (54.0%)	61 (52.1%)	58 (54.2%)	43 (56.6%)	0.832
*Yes*	138 (46.0%)	56 (47.9%)	49 (45.8%)	33 (43.4%)	
**R Glycopentide**					
*No*	149 (49.7%)	50 (42.7%)	59 (55.1%)	40 (52.6%)	0.150
*Yes*	151 (50.3%)	67 (57.3%)	48 (44.9%)	36 (47.4%)	
**R Daptomicina**					
*No*	170 (56.7%)	61 (52.1%)	64 (59.8%)	45 (59.2%)	0.447
*Yes*	130 (43.3%)	56 (47.9%)	43 (40.2%)	31 (40.8%)	
**R Fluorochinoloni**					
*No*	87 (29.0%)	33 (28.2%)	30 (28.0%)	24 (31.6%)	0.848
*Yes*	213 (71.0%)	84 (71.8%)	77 (72.0%)	52 (68.4%)	
**R Fosfomicina**					
*No*	236 (78.7%)	93 (79.5%)	80 (74.8%)	63 (82.9%)	0.401
*Yes*	64 (21.3%)	24 (20.5%)	27 (25.2%)	13 (17.1%)	
**R Colistina**					
*No*	132 (44.0%)	42 (35.9%)	44 (41.1%)	46 (60.5%)	0.003
*Yes*	168 (56.0%)	75 (64.1%)^*^	63 (58.9%)^*^	30 (39.5%)	
**R Linezolid**					
*No*	175 (58.3%)	60 (51.3%)	65 (60.7%)	50 (65.8%)	0.111
*Yes*	125 (41.7%)	57 (48.7%)	42 (39.3%)	26 (34.2%)	

*N* (%) are shown when appropriate. *p*-value < α for Pearson’s chi-square test between couples: *S. capitis* (Np vs. Pp-COVID); peripheral blood cultures (Pp-non-COVID vs. Np and Pp-COVID vs. Np); CVC blood cultures (Pp-non-COVID vs. Np and Pp-COVID vs. Np); Oxa R (Pp-non-COVID vs. Np and Pp-COVID vs. Np); ESBL (Pp-COVID vs. Np); R Carbapenemi (Pp-COVID vs. Np); R Colistina (Np vs. Pp-COVID and Pp-non-COVID vs. Pp-COVID).

About germs, there was no significant difference in germ circulation between Gram-positive vs. Gram-negative among groups (Np 58.1%, Pp-noCOVID 59.8%, Pp-COVID 61.8% vs. Np 41.9%, Pp-noCOVID 40.2%, Pp-COVID 38.2%, *p* = 0.875, respectively). Between the Np vs. Pp period, we observed an increase in a few Gram-positive bacteria, such as *S. capitis* (1 patient 0.9% vs. 14 patients 7.65%, *p* = 0.008), *S. epidermidis, Streptococcus* spp.*, and E. faecalis* and a decrease in cases of blood culture positive for *S. aureus, S. hominis, and E. faecium.*

Finally, in Gram-negative bacteria, we observed an increase in cases of *Acinetobacter* spp. (Np 6 pt. −5.1%- vs. Pp 20 pt. −10.9%, *p* = 0.082) and *Serratia* spp., while the cases of sepsis decreased from *E. faecium* (Np 11 pt. −9.4%- vs. Pp 7 pt. −3.8%, *p* = 0.047), and *Enterobacter* spp., *S. haemolyticus*, *S. maltophilia*, *Proteus* spp., and *P. aeruginosa* have not changed over the time of the study.

## Discussion

We found a substantial increase in the prevalence of cases of sepsis and septic shock in patients admitted to the ICU from 2018 to 2020 and a reduction in 2021.

Sepsis is characterized by a high mortality rate. The rate estimates range from 10 to 52%, depending on how the data are collected (16–20). Mortality rates increase linearly according to the severity of the disease (19). In one study, the mortality rates of septic shock were 46% (21). In another study, the mortality associated with sepsis was ≥10%, while in the case of septic shock, it was ≥40% (22). We did not detect differences in the medical or surgical origin of sepsis in the study periods, probably due to the sample size. In addition, we know that patients with sepsis and positive blood cultures have a higher severity of illness and higher mortality (23) and this represents a particular group risk population.

There are different studies on patients in ICU with a mortality rate of 59% in COVID-19 sepsis vs. 29% in the same period without COVID-19, or 58.7% vs. 40%, respectively (24, 25). Our data show that in ICU patients, there was an increase in the 30-day mortality rate from 2018 to 2020, with a reduction in 2021 and a return to mortality values in the pre-COVID-19 era. This trend can probably be traced back to the different phases of the COVID-19 disease that impacted patient survival. Indeed, in 2020, there was a pandemic that caught healthcare systems unprepared with the absence of knowledge related to COVID-19 physiopathology and its treatment. Initially, the physiopathology of the SARS-CoV-2 infection was unknown. COVID-19 is classically divided into two phases: the first is characterized by a high viral load, while the second is associated with the activation of an inflammatory response, including the appearance of a cytokine storm, which is then responsible for the evolution of ARDS and MOF, and eventually death (3, 26, 27).

Numerous therapies were attempted in the first half of 2020 that proved largely ineffective. Only in the second half of 2020 did pathophysiological knowledge increase, and the discovery of effective therapeutic strategies made it possible to approach patients better, allowing for better survival even in patients with septic complications, as can be seen from the 2021 data (27–29).

The subsequent diffusion of the massive vaccination strategy resulted in the modification of the severity of COVID-19, allowing for the development of vaccine immunity, which changed the natural history of the disease due to a more ready immune system response to infection. Finally, the greater availability and increasingly correct use of DPI have probably contributed to the reduction of the incidence of sepsis and mortality in patients observed in 2021.

There are no clear data on mortality rates in the COVID-19 era, but in particular, there are no data on the mortality rates of patients in the ICU who died from sepsis and SARS-CoV-2 infection.

In the general population, COVID-19-related mortality appears to be lower in younger patients (<44 years) without comorbidities (<10%) (30). Risk factors for mortality are known in COVID-19 and sepsis, such as advancing age, immunosuppression, and hospitalization (31, 32). This concordance of factors could help explain why we have seen an increase in sepsis in the first phase of COVID-19.

In this ICU population, age is a significant risk factor for mortality; data about this are widely available in the literature (33, 34). Another risk factor highlighted in our study is the correlation between mortality and days of stay in the ICU. Furthermore, as expected, other risk factors associated with mortality included comorbidities, which affected fewer COVID-19 patients than non-COVID-19 patients in this study. Patients with sepsis who were diagnosed with COVID-19 had fewer comorbidities. These data are attributable to the fact that patients with SARS-CoV-2 infection were mainly hospitalized for severe respiratory insufficiency, while, as is known, patients admitted to intensive care without a diagnosis of COVID-19 were hospitalized for the appearance of septic shock, which we know is linked to the presence of comorbidities. In this study with septic patients, the SOFA and APACHE scores were correlated with mortality, but the SOFA score is higher in COVID-19 patients vs. non-COVID-19 patients. These data can also be explained by the clinical conditions that were secondary to the cytokine storm that evolves in ARDS or MOF.

Neutrophils are the first immune cells recruited to the site of inflammation following stimulation by chemotactic factors released from damaged pulmonary tissues. Both exogenous and endogenous inflammatory stimuli can be recognized by specific receptors in human neutrophils. This further promotes the recruitment and activation of circulating neutrophils. These activated neutrophils produce several cytotoxic products and various proinflammatory cytokines. The overwhelming activation of neutrophils contributes to surrounding tissue damage and even lung dysfunction (35). Therefore, in COVID-19 ARDS patients, higher counts of neutrophils are observed and represent a predictor of poor outcome (36, 37).

A previous study on COVID-19 patients showed that neutrophils are an early marker in high-risk COVID-19 patients for acute respiratory failure and organ damage. Based on these results, we believe that classic inflammation markers such as CRP are not sufficient for stratification on COVID-19 patients. Instead, the dosing of factors among the relationship between neutrophils and lymphocytes (NRLs), IL-6, LDH, and ferritin could be useful for the early identification of patients at high risk of ARDS and death (3, 36).

Furthermore, in our study, it emerged that in the pre-pandemic era, the cases of sepsis associated with blood culture from a peripheral vein were statistically higher, while in the COVID-19 period, the cases of sepsis isolated from CVC increased. These data have never been found in the literature, and the reasons for this increased incidence of CVC-related infections could be associated with increased use of CVC and immunosuppression secondary to SARS-CoV-2 infection and to the cytokine storm phase that makes the immune system particularly dysregulated (27).

In the literature, we know that the types of sepsis-related microorganisms have changed over time. Gram-positive bacteria are mostly responsible for sepsis in the United States, although the number of cases of Gram-negative sepsis remains remarkable (32, 38). The incidence of fungal sepsis has increased over the past decade but remains lower than bacterial sepsis (16). In approximately one-half of cases of sepsis, the microorganism is not identified, so we have culture-negative sepsis (39). In our study, we have highlighted in the pandemic era a significant increase in cases of sepsis from the *CNS and Acinetobacter* spp. The epidemiological report of the European Centre for Disease Prevention and Control (ECDC) on hospital-acquired infections in the ICU, computed from 2017 data, showed a predominance of Gram-positive pathogens in bloodstream infections (40). Gram-negative bacilli cause approximately a quarter to a half of all bloodstream infections, and this depends on geographic region, whether the onset of the infection is in the hospital or the community, and other patient risk factors (41). This study showed an increase in *S. capitis* and *S. epidermidis*, but also in Gram-negative bacteria such as *Acinetobacter* spp. and *Serratia* spp. These data agree with the data relating to germs usually circulating in the ICU, but they do not seem to agree with a recent study in Iraq that instead shows a high incidence of Gram-positive sepsis mainly caused by *Streptococcus, Haemophilus,* and *Moraxella* (42). Probably, the different circulation of Gram-positive bacteria is related to the different characteristics of the patients and the countries.

We observed a significant reduction in the number of resistances of isolated germs, which may also be linked to a reduced selective pressure of antibiotic therapy for a better and more appropriate use of antibiotic therapy especially in the COVID-19 period. Patients in the ICU frequently are on or have recently been on antibiotics, which increases the risk of infections with *P. aeruginosa* and other non-fermenting Gram-negative *bacilli*, such as *Acinetobacter species*, that have intrinsic or acquired resistance to commonly used agents.

In our study, we particularly observed over time during the pandemic era a decreased resistance related to OXA-48, ESBL, carbapenems, and colistin. Our data contrast with unique but recently published data on non-ICU patients showing a higher incidence of ESBL-producing *E. coli* in COVID-19 patients than in non-COVID-19 patients. ESBL infections are associated with longer hospital stays and higher mortality rates in different population situations (43); these data are probably linked to a decrease in the number of Gram-negative bacteria that also led to the reduction of ESBLs.

In agreement with the literature data, we found a significant correlation between mortality from sepsis or septic shock and the presence of an ESBL germ. Neither was associated with the APACHE score and therefore with the patient’s clinical severity or with the diagnosis of COVID-19 (44, 45).

We know that the limitations of this study are the lack of information on colonization rates and molecular analysis for clusters of bacteria isolates. Additionally, our study is a single-center study, and therefore, our results cannot be generalized to all conditions. Perhaps during the pandemic phase, there was a more appropriate use of antibiotic therapies, but above all, PPE was used more correctly in ICUs.

## Conclusion

This study demonstrated how the COVID-19 pandemic changed the prevalence of sepsis in the ICU. It emerged that the risk factors associated with mortality were SOFA and APACHE scores, age, days in the ICU, and, above all, the presence of ESBL-producing bacteria. Despite this, during the pandemic phase, we have observed a significant reduction in the emergence of resistant germs compared to the pre-pandemic phase. The COVID-19 pandemic revealed the critical need for effective infection-control policies and the correct use of antibiotic stewardship, along with a number of interventions to reduce sepsis and other co-infections in COVID-19 units. Compliance with guidelines for infection control and standards of antibiotic care is imperative.

## Data availability statement

The original contributions presented in the study are included in the article/supplementary material, further inquiries can be directed to the corresponding author.

## Ethics statement

The study protocol was approved by the Ethics Internal Committee at the University “G. d’Annunzio” ChietiPescara (Ethics Committee Project No. 02 the 02/02/2022) and was performed in accordance with the ethical standards laid down in the 1964 Declaration of Helsinki. Written informed consent from the patients was not required to participate in this study in accordance with the national legislation and the institutional requirements.

## Author contributions

KF: Conceptualization, Writing – original draft. LV: Data curation, Validation, Writing – original draft. PB: Data curation, Formal analysis, Writing – review & editing. MN: Data curation, Formal analysis, Writing – review & editing. CU: Conceptualization, Funding acquisition, Writing – review & editing. AG: Funding acquisition, Methodology, Writing – review & editing. MB: Investigation, Methodology, Writing – review & editing. GT: Data curation, Methodology, Resources, Writing – review & editing. JV: Conceptualization, Supervision, Writing – original draft. SM: Conceptualization, Supervision, Writing – original draft.
